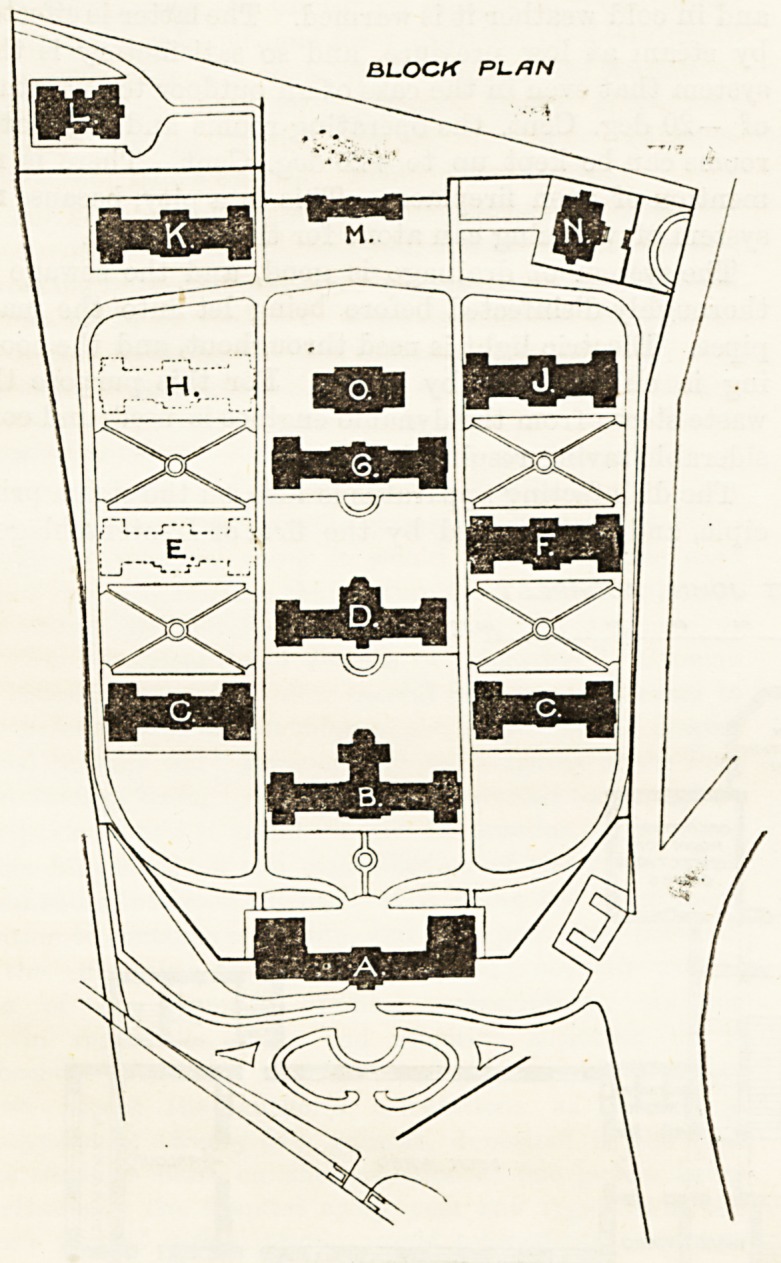# Hospital Construction

**Published:** 1899-11-18

**Authors:** 


### The Institutional Workshop.

### HOSPITAL CONSTRUCTION.

### THE HOSPITAL OF ST JOHN, BUDA-PEST.

Buda-Pest is the name given to the capital town of Hungary. It is situated on the Danube, about 130 miles from Vienna. There are really two towns, Buda being on the right bank of the river, and Pest (or Pesth) on the left bank. They are connected by a fine suspension bridge. The population is about 500,000.

Here has been lately built one of the finest hospitals in Europe. It was designed by Dr. A. Ludvik, the director of all the hospitals on the right side of the Danube, and who is also one of the leading surgeons of the country.

The hospital is built on a delightful site on the outskirts of the town, and is surrounded with green hills and trees. The pavilion system has been adopted, and when the whole hospital is finished there will be 15 blocks entirely detached. This system ensures rather freer circulation of air among the blocks, but it increases the labour and difficulty of supervision; and we should have preferred a one-storey corridor at either end of the central row of blocks, and connecting the side blocks. The corridors would occupy almost the same position as the straight terraces now do.

The buildings cover an area of upwards of 60,000 square yards, and they are about 170 ft. above the level of the Danube. The hospital blocks contain at present 725 beds. The arrangement will be understood by noting that A is the administration block; B, the surgical pavilion; C C, internal diseases; D, nurses' rooms; E, convalescent block being erected; F, obstetric block; G, kitchens; H, future extension; I, diseases of children; K, ophthalmic diseases; L, infectious diseases; M, stores; N, mortuary block; and O, engine-room. The longitudinal axes of the blocks lie north and south, so that the prevailing winds will strike their ends. The basements of the blocks are devoted entirely to the arrangements for warming and ventilating. Before being supplied to the ward the air is freed from dust, and in cold weather it is warmed. The latter is effected by steam at low pressure, and so satisfactory is this system that even in the case of an outdoor temperature of −20 deg. Cent. the operating-rooms and the bathrooms can be kept up to +25 deg. Cent. There is no mention of open fireplaces. This is a pity, because no system of warming can atone for their absence.

The system of drainage is good, and the sewage is thoroughly disinfected before being let into the main pipes. Electric light is used throughout, and the cooking is chiefly done by steam. For this purpose the waste steam from the dynamo engines is used, and considerable saving results thereby.

The disinfecting apparatus is also on the steam principle, and was supplied by the firm of Lautenschläger, of Berlin. The total cost of the hospital and its adjuncts exceeded £120,000.

THE NEW HOSPITAL OF ST JOHN, BUDAPEST.

SURGICAL PAVILION
GROUND PLAN

In addition to the block plan already described, we give the ground plan of the surgical pavilion. It consists of a centre and two wings. The latter contains the main entrance, three small wards, medical officers' rooms, operation-room for infectious cases, preparation-room, and the general operating-room. This is the chief feature of the block. It is octagonal in shape, constructed entirely of glass and iron. The glass walls are double, and curtains are placed between the sheets of glass so that the light can be controlled at will. Every operating-room should have overhead light as well as side light, and this has not been forgotten here. There is a complete Röntgen laboratory.

The large dormitories are placed in the wings, and they are intended for 18 beds each. They are nearly 60 ft. long, 29 ft. wide, and 16 ft. high. At the end of each dormitory is a parlour or day-room opening into a verandah or balcony. This is a very pleasant feature. The bath-room and scullery project from one side.

and the closets and wardrobe from the other. A great mistake, or what would certainly be considered a great mistake in England, has been made in not having a ventilating passage between the closets and the ward.

We are indebted to Dr. Charles Bodon for the plans and the notes from which this description has been written.

## Figures and Tables

**Figure f1:**
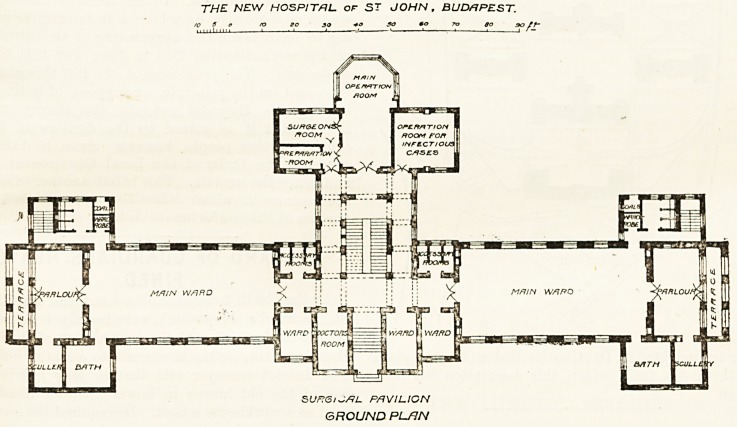


**Figure f2:**